# 2D Octagon-Structure Carbon and Its Polarization Resolved Raman Spectra

**DOI:** 10.3390/nano10112252

**Published:** 2020-11-13

**Authors:** Chunshan He, Weiliang Wang

**Affiliations:** 1 State Key Laboratory of Optoelectronic Materials and Technologies, School of Physics, Sun Yat-sen University, Guangzhou 510275, China; stshcs@mail.sysu.edu.cn; 2 State Key Laboratory of Optoelectronic Materials and Technologies, School of Physics, Guangdong Province Key Laboratory of Display Material and Technology, Sun Yat-sen University, Guangzhou 510275, China

**Keywords:** polarized Raman, vibrational properties, 2D carbon, DFT

## Abstract

We predict a new phase of two-dimensional carbon with density functional theory (DFT). It was found to be semimetal with two Dirac points. The vibrational properties and the polarization resolved Raman spectra of the carbon monolayer are predicted. There are five Raman active modes: 574 cm^−1^ (E_g_), 1112 cm^−1^ (B_1g_), 1186 cm^−1^ (B_2g_), 1605 cm^−1^ (B_2g_) and 1734 cm^−1^ (A_1g_). We consider the incident light wave vector to be perpendicular and parallel to the plane of the carbon monolayer. By calculating Raman tensor of each Raman active mode, we obtained polarization angle dependent Raman intensities. Our results will help materials scientists to identify the existence and orientation of octagon-structure carbon monolayer when they are growing it.

## 1. Introduction

Graphene has become a hot scientific topic since 2004 [1]. A lot of theoretical and experimental results show that graphene has remarkable properties in mechanics, thermology, electronics and optics [2,3]. In recent years, people found many 2D materials from allotropes of carbon and from group V elements. Some of them have potential application in optoelectronic, nano electronic devices and hydrogen storage, because of their unique electronic and mechanical properties [4,5,6,7,8,9,10]. In 2018, Cao et al. found the twisted bilayer graphene is able to be an insulator or superconductor [11,12]. In the last few decades, scientists have sought and researched some other 2D materials for nanoelectronic, optoelectronic and thermoelectronic devices [13], such as hexagonal boron nitride (*h*-BN) [14,15,16] and transition metal dichalcogenides (TMDCs) [17,18,19,20,21,22]. Zhang et al. predicted a novel 2D octagon-structure monolayer of nitrogen [23]. Its structure is composed of squares and octagons. There are many good electronic properties of this 2D material. It could be a potential candidate for use in the semiconductor devices, spintronics and quantum computation. Octagon-structure materials can be expected to be a good candidate for hydrogen storage. It is a possible high-temperature superconductor [24]. In 2015, Lu et al. fabricated free-standing octagon-shaped carbon nanofiber [25]. In 2017, Zhong’s research group fabricated graphene-like nanoribbons which are periodically embedded with four- and eight-membered rings [26].

In this work, we theoretically predict a new phase of 2D carbon which consists of octagon carbon rings. Raman spectroscopy is a widely used experimental tool to characterize materials without destroying the samples [27,28]. People observed polarization dependent Raman intensity in many 2D materials, such as WS_2_ and MoS_2_ [29,30,31,32]_._ Therefore, we investigated the vibrational properties and predict the Raman spectra of this 2D octagon-structure carbon by density functional theory (DFT) to help materials scientists to identify the existence of this new phase of 2D carbon.

In 2011, a carbon allotrope named as T-carbon was predicted by Su and his co-workers in theory [33]. Six year later, Zhang et al. synthesized T-carbon from pseudo-topotactic conversion of a multi-walled carbon nanotube suspension in methanol by picosecond pulsed-laser irradiation [34]. Therefore, the 2D Octagon-structure Carbon is holds much hope for synthesis in the future. This work sets a goal for materials scientists, providing a way for them to verify their experimental results, and providing a vast playground for theorists.

## 2. Materials and Methods

Geometry optimization, force constant calculation and dielectric tensor calculation were performed with the Vienna Ab initio simulation package (VASP, version 5.4.4., Wien, Austria) [35]. Phonon wavenumber and phonon modes were obtained with the Phonopy code [36]. The electron-core interactions were treated in the projector augmented wave (PAW) approximation [35,37,38,39]. The density functional was treated by generalized gradient approximation (GGA) with the Perdew–Burke–Ernzerhof (PBE) exchange correlation potential [40]. Kinetic energy cutoffs of 520 eV and 400 eV were used in geometry optimization and other calculations, respectively. The *k* point mesh was 15 × 15 × 1, 20 × 20 × 1 and 51 × 51 × 1 in geometry optimization, force constant calculation and dielectric function calculation, respectively. Vacuum slabs of 5 nm thick were inserted between neighboring 2D atom sheets. The convergence tolerance for the total energy and force calculations were set to 10^−8^ eV and 10^−5^ eV/ Å, respectively.

The Raman tensor was obtained with the finite displacement method [31,32,41,42,43,44,45,46,47,48,49,50,51,52]:

(1) $$R_{\alpha \beta }\left(j\right)=V\overset{N}{\underset{\mu =1}{\sum }}\overset{3}{\underset{l=1}{\sum }}\frac{\partial \chi_{\alpha \beta }}{\partial r_{l}\left(\mu \right)}\frac{e_{l}^{j}\left(\mu \right)}{\sqrt{M_{\mu }}}$$

where *V* is the volume of unit cell, *M_μ_* is the atomic mass of atom *μ*, ∂*χ_αβ_*/∂*r_l_*(*μ*) is the first derivative of the electric polarizability tensor with respect to the atomic displacement. It equals the derivative of dielectric tensor *ε_αβ_* divided by 4*π* because *χ_αβ_*(*ω*) = (*ε_αβ_*(*ω*) − *δ_αβ_*)/4*π*. *ω* is the frequency of the laser (electric field). *e^j^_l_*(*μ*) is the eigenvector of the j-th phonon mode at Г point. The dielectric tensor of the structure with finite displacement can be obtained with DFT.

Then Raman intensity can be obtained with [31,32]:

(2) $$I\propto \omega_{s}^{4}{\left|{\vec{e}}_{s}\cdot \overset{↔}{R}\cdot {\vec{e}}_{i}\right|}^{2}$$

where $\omega_{s}$ is the frequency of scattered light; ${\vec{e}}_{i}$ and ${\vec{e}}_{s}$ are electric polarization vectors of incident and scattered light, respectively.

## 3. Results

**Figure 1:**
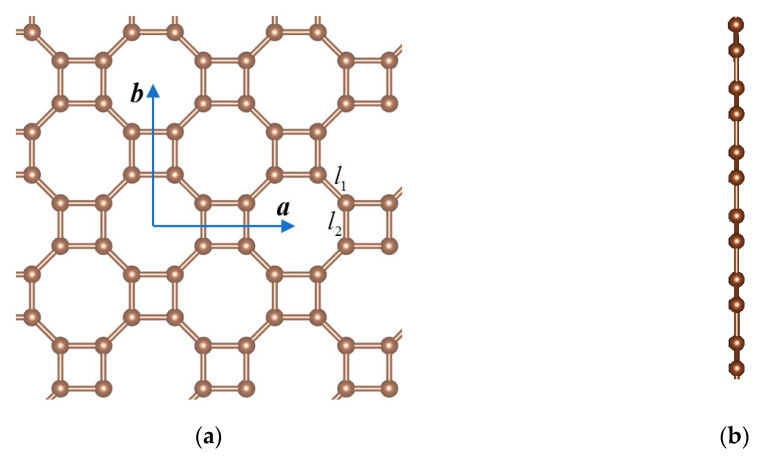
(**a**) Top view and (**b**) side view of the octagon-structure carbon monolayer. The blue arrows ***a*** and ***b*** show two basis vectors of the unit cell which are along the *x*-axis and *y*-axis, respectively. The bond lengths *l*_1_ and *l*_2_ are the short and long edges of the octagons.

**Figure 2:**
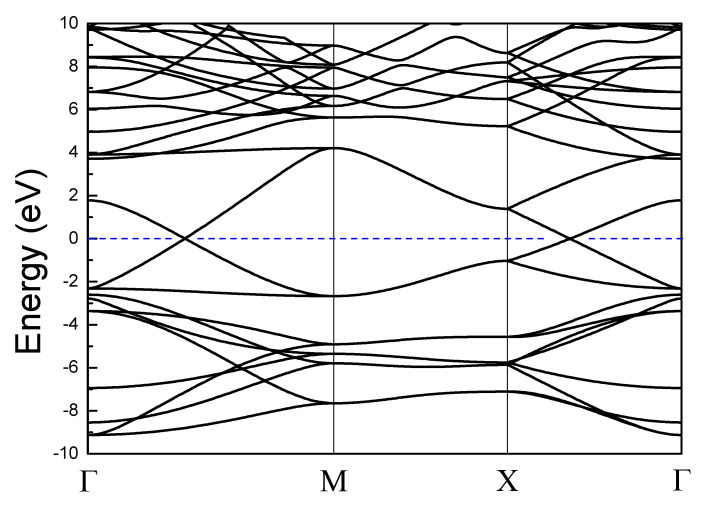
Band structure of the octagon-structure carbon monolayer.

The unit cell of monolayer octagon-structure carbon consists of eight atoms (Figure 1a). The lattice constants: *a* = *b* = 4.87 Å; the bond length *l*_1_ = 1.373 Å, *l*_2_ = 1.466 Å. The buckled displacement Δz is zero (Figure 1b). It belongs to space group P4/mmm (123). The stable lattice structures are shown in Figure 1. Figure 2 shows its band energy structures. It is a semimetal with two Dirac points.

**Figure 3:**
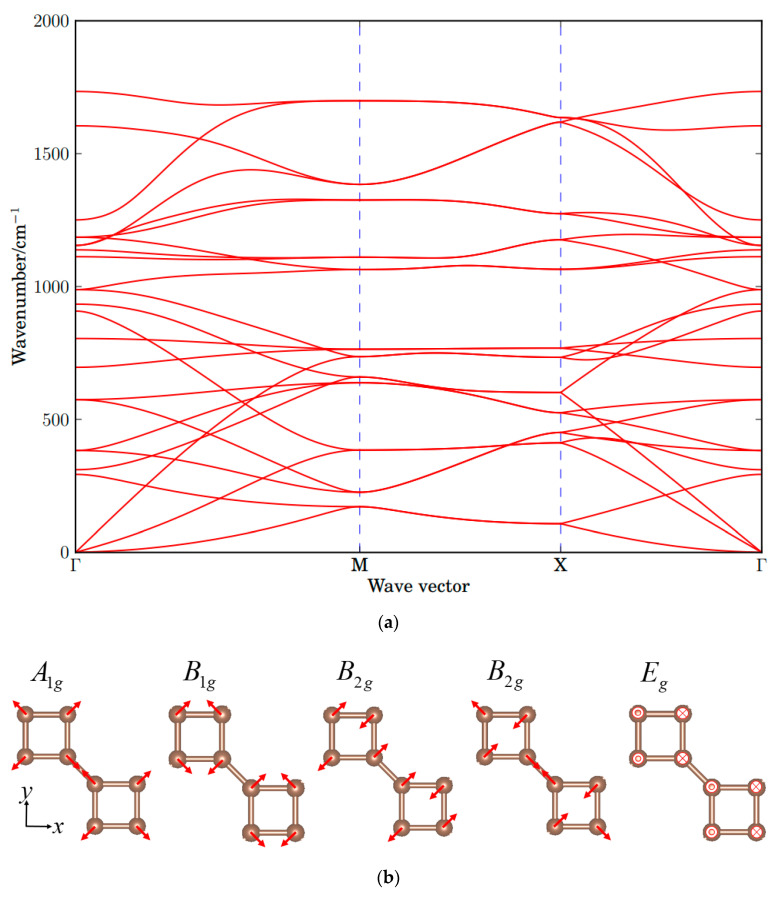
(**a**) Phonon dispersions of the octagon-structure carbon monolayer, (**b**) Raman active modes A_1g_ (in-plane), B_1g_ (in-plane), B_2g_ (in-plane) and E_g_ (out-of-plane) at Γ point. The dot and cross on the atoms refer to displacement in and out of the plane, respectively, and the red arrows refer to the in-plane displacement. The length of the arrows is nonphysical.

Figure 3a is the phonon dispersion of the carbon monolayer. It does not have imaginary vibrating modes. The octagon-structure carbon monolayer belongs to the D_4h_ point group, whose Raman active modes are A_1g_ (in-plane vibration), B_1g_ (in-plane vibration), B_2g_ (in-plane vibration) and E_g_ (out-of-plane vibration). The atomic vibration displacements are shown in Figure 3b. The Γ point wavenumbers of the A_1g_ mode and B_1g_ mode are 1734 cm^−1^ and 1112 cm^−1^, respectively. The Γ point wavenumbers of the two B_2g_ modes are 1186 and 1605 cm^−1^. The Γ point wavenumber of the E_g_ mode is 574 cm^−1^.

In order to calculate the Raman intensity, we chose three commonly used laser lines whose wavelengths are 488 nm (2.54 eV), 532 nm (2.33 eV) and 633 nm (1.96 eV) [27,30]. The coordinate system is chosen so that the monolayer plane is in the *x*-*y* plane. The calculated results show that the Raman tensors agree with the point group D_4h_ and their irreducible representations. They are the following matrix forms:

(3) $$\tilde{R}(A_{1g})=\left(\begin{array}{ccc} a & 0 & 0\\ 0 & a & 0\\ 0 & 0 & b \end{array}\right)\;\tilde{R}(B_{1g})=\left(\begin{array}{ccc} c & 0 & 0\\ 0 & -c & 0\\ 0 & 0 & 0 \end{array}\right)\;\tilde{R}(B_{2g})=\left(\begin{array}{ccc} 0 & d & 0\\ d & 0 & 0\\ 0 & 0 & 0 \end{array}\right)\;\tilde{R}(E_{g})=\left(\begin{array}{ccc} 0 & 0 & e\\ 0 & 0 & f\\ e & f & 0 \end{array}\right)$$

where *a*, *b*, *c*, *d*, *e*, *f* are complex numbers.

When the incident light wave vector is perpendicular to the monolayer plane (*x*-*y* plane), the polarization direction of the incident light is in the *x*-*y* plane and can be written as $e_{i}=(\cos\theta ,\sin\theta ,0)$. We considered two polarization directions of scattered light which are parallel and perpendicular to the polarization direction of the incident light, called parallel polarization configuration and perpendicular polarization configuration, respectively. Then the polarization directions of the scattered light can be written as $e_{s}^{∥}=(\cos\theta ,\sin\theta ,0)$ and $e_{s}^{⊥}=(-\sin\theta ,\cos\theta ,0)$, respectively. Based on the Raman tensors matrix and polarization directions of the incident and scattered light, we can obtain the polarization angle dependent Raman intensity for each mode. The Raman intensities for these polarization configurations are:

(4) $$I^{∥}\left(A_{1g}\right)\propto {\left|a\right|}^{2}$$

(5) $$I^{∥}\left(B_{1g}\right)\propto {\left|c\right|}^{2}\cos^{2}(2\theta )$$

(6) $$I^{∥}\left(B_{2g}\right)\propto {\left|d\right|}^{2}\sin^{2}\left(2\theta \right)$$

(7) $$I^{∥}\left(E_{g}\right)\propto 0$$

(8) $$I^{⊥}\left(A_{1g}\right)\propto 0$$

(9) $$I^{⊥}\left(B_{1g}\right)\propto {\left|c\right|}^{2}\sin^{2}\left(2\theta \right)$$

(10) $$I^{⊥}\left(B_{2g}\right)\propto {\left|d\right|}^{2}\cos^{2}\left(2\theta \right)$$

(11) $$I^{⊥}\left(E_{g}\right)\propto 0$$

**Figure 4:**
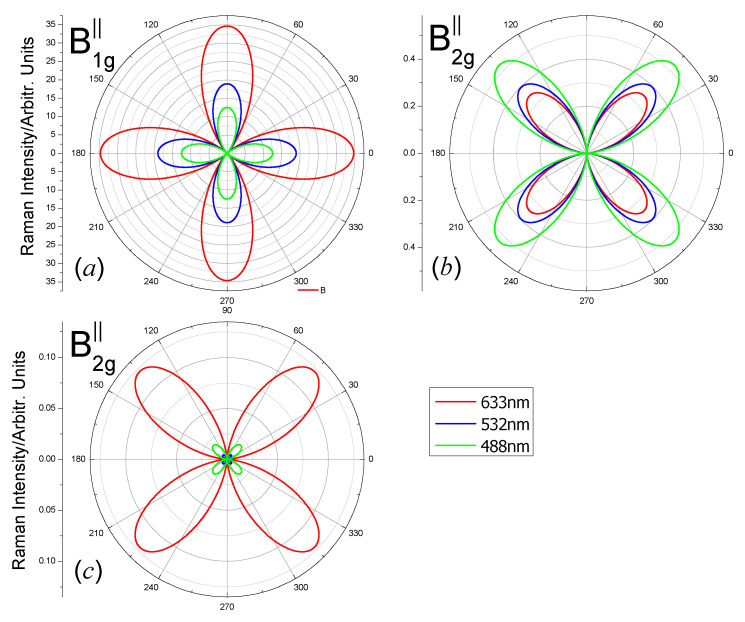
Polar plots of the angular dependent Raman intensities ${\left|{\vec{e}}_{s}\cdot \overset{↔}{R}\cdot {\vec{e}}_{i}\right|}^{2}$ of the (**a**) B_1g_-1112 cm^−1^, (**b**) B_2g_-1605 cm^−1^ and (**c**) B_2g_-1186 cm^−1^ modes of the octagon-structure carbon monolayer excited by laser lines 488 nm (green), 532 nm (blue) and 633 nm (red). The polarization direction of the scattered light is parallel to the polarization direction of the incident light. The incident and scattered light wave vectors are in the *z*-direction.

**Figure 5:**
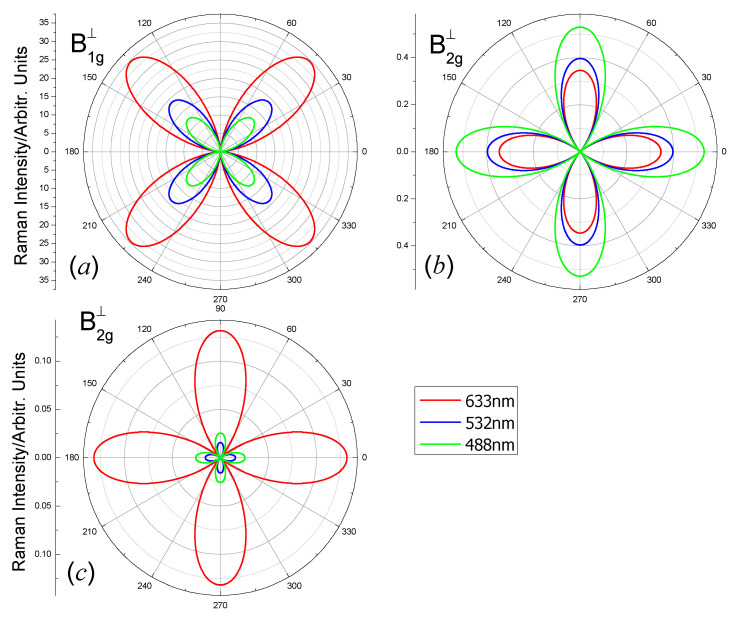
Polar plots of the angular dependent Raman intensities ${\left|{\vec{e}}_{s}\cdot \overset{↔}{R}\cdot {\vec{e}}_{i}\right|}^{2}$ of (**a**) B_1g_-1112 cm^−1^, (**b**) B_2g_-1605 cm^−1^ and (**c**) B_2g_-1186 cm^−1^ modes of the octagon-structure carbon monolayer excited by laser lines 488 nm (green), 532 nm (blue) and 633 nm (red). The polarization direction of the scattered light is perpendicular to the polarization direction of the incident light. The incident and scattered light wave vectors are in the *z*-direction.

For the A_1g_ mode, Equations (4) and (8) mean that the scattered lights intensity is independent of the polarization angle θ. The intensity is zero for the E_g_ mode. Only the B_1g_ modes and B_2g_ modes show the polarization angle dependent intensity. The polarization dependent Raman intensities of the B_1g_ modes and B_2g_ modes of the carbon monolayer for three laser lines 488 nm, 532 nm and 633 nm, when the wave vector of the laser is perpendicular to the 2D plane, are shown in Figure 4 (parallel polarization configuration) and Figure 5 (perpendicular polarization configuration). The order of magnitude of Raman intensities excited by different laser lines is different because the Raman intensity is proportional to $\omega_{s}^{4}$ (Equation (2)). Therefore, we plot ${\left|{\vec{e}}_{s}\cdot \overset{↔}{R}\cdot {\vec{e}}_{i}\right|}^{2}$ for clarity.

From Figure 4, Raman intensities of the B_1g_ and B_2g_ modes in parallel polarization configurations have four maxima and four minima for all the laser lines. Due to the factor $cos^{2}\left(2\theta \right)$ in Equation (5), the maxima of Raman intensities of B_1g_ modes (Figure 4a) locate at *θ* = 0° + *n* × 90° (*n* = 0, 1, 2, 3); the four minima locate at *θ* = 45° + *n* × 90° (*n* = 0, 1, 2, 3). Due to the factor $sin^{2}\left(2\theta \right)$ in Equation (6), the maxima of Raman intensities of B_2g_ modes (Figure 4b,c) locate at *θ* = 45° + *n* × 90° (*n* = 0, 1, 2, 3); the four minima locate at *θ* = 0° + *n* × 90° (*n* = 0, 1, 2, 3). The location of the maxima and minima in Figure 5 are result from a similar reason to that in Figure 4. The polarization angle dependent Raman intensity with the incident light wave vector in the z direction is different from some other isotropic 2D materials (e.g., *β*-Arsenic monolayer [47] and MXenes Zr_2_X(X = C and N) [49]). The anisotropic vibrations are responsible for the polarization dependent Raman intensity.

If the incident light wave vector is along the *x*-axis, the polarization direction of the incident light is in the *y*-*z* plane and can be written as $e_{i}=(0,\cos\theta ,\sin\theta )$. We also considered parallel polarization configuration and perpendicular polarization configuration, in which the polarization directions of the scattered light can be written as $e_{s}^{\left|\right|}=(0,\cos\theta ,\sin\theta )$ and $e_{s}^{⊥}=(0,-\sin\theta ,\cos\theta )$, respectively. Based on the Raman tensors matrix and polarization directions of the incident light and scattered light, we can obtain the polarization angle dependent Raman intensity for each mode. The Raman intensities are:

(12) I∥(A1g)∝|a|2cos4θ+|b|2sin4θ+Re(a·b*)sin2(2θ)/2

(13) $$I^{∥}\left(B_{1g}\right)\propto {\left|c\right|}^{2}\cos^{4}\theta$$

(14) $$I^{∥}\left(B_{2g}\right)\propto 0$$

(15) $$I^{∥}\left(E_{g}\right)\propto {\left|f\right|}^{2}\sin^{2}\left(2\theta \right)$$

(16) I⊥(A1g)∝[|a|2+|b|2−2Re(a·b*)]sin2(2θ)/4

(17) $$I^{⊥}\left(B_{1g}\right)\propto {\left|c\right|}^{2}\sin^{2}\left(2\theta \right)/4$$

(18) $$I^{⊥}\left(B_{2g}\right)\propto 0$$

(19) $$I^{⊥}\left(E_{g}\right)\propto {\left|f\right|}^{2}\cos^{2}\left(2\theta \right)$$

**Figure 6:**
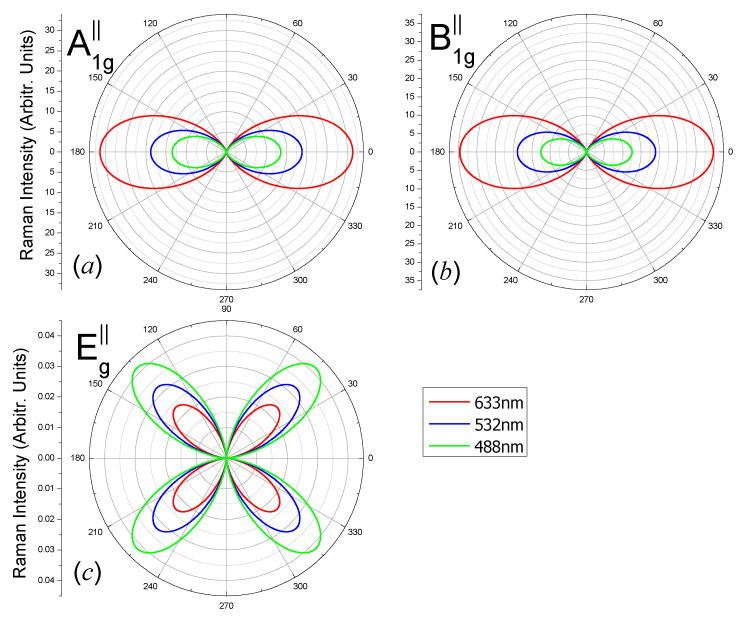
Polar plots of the angular dependent Raman intensities ${\left|{\vec{e}}_{s}\cdot \overset{↔}{R}\cdot {\vec{e}}_{i}\right|}^{2}$ of (**a**) A_1g_-1734 cm^−1^, (**b**) B_1g_-1112 cm^−1^ and (**c**) E_g_-574 cm^−1^ modes of the octagon-structure carbon monolayer excited by laser lines 488 nm (green), 532 nm (blue) and 633 nm (red). The polarization direction of the scattered light is parallel to the polarization direction of the incident light. The incident and scattered light wave vectors are in the *x*-direction.

**Figure 7:**
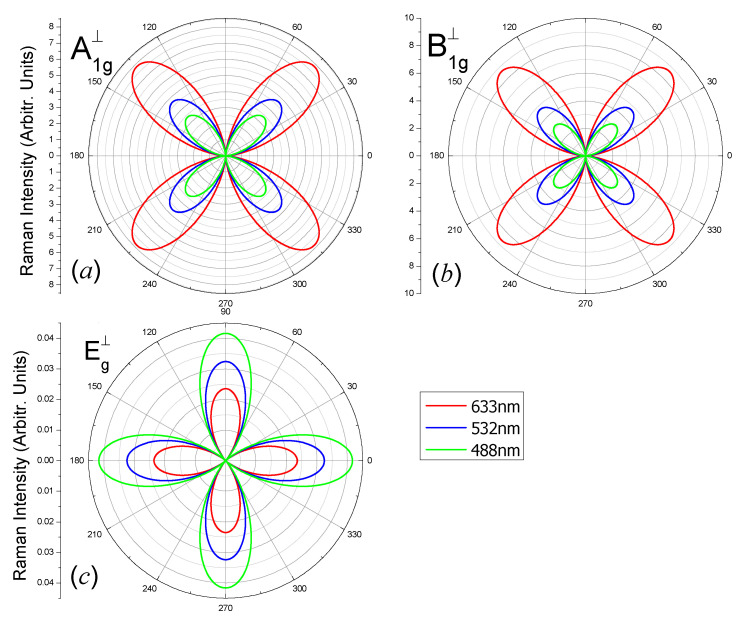
Polar plots of the angular dependent Raman intensities ${\left|{\vec{e}}_{s}\cdot \overset{↔}{R}\cdot {\vec{e}}_{i}\right|}^{2}$ of (**a**) A_1g_-1734 cm^−1^, (**b**) B_1g_-1112 cm^−1^ and (**c**) E_g_-574 cm^−1^ modes of the octagon-structure carbon monolayer excited by laser lines 488 nm (green), 532 nm (blue) and 633 nm (red). The polarization direction of the scattered light is perpendicular to the polarization direction of the incident light. The incident and scattered light wave vectors are in the *x*-direction.

The angular dependent Raman intensities of different vibrating modes of the carbon monolayer for three laser lines 488 nm, 532 nm and 633 nm, when the wave vector is parallel to the 2D plane, are shown in Figure 6 (parallel polarization configuration) and Figure 7 (perpendicular polarization configuration).

From Figure 6, the maxima locate at 0° and 180° for the A_1g_ modes (Figure 6a), because |*a*| is always much larger than |*b*|, which may change with laser photon energy; the maxima locate at 0° and 180° for B_1g_ modes (Figure 6b) due to the factor $cos^{4}\theta$; the Raman intensities have four maxima which locate at θ = 45° + *n* × 90° (*n* = 0, 1, 2, 3) for the E_g_ mode (Figure 6c) due to the factor $sin^{2}\left(2\theta \right)$.

From Figure 7a,b, Raman intensities of the A_1g_ and B_1g_ modes in perpendicular polarization configurations have four maxima for all the laser lines; the maxima intensities locate at *θ* = 45° + *n* × 90° (*n* = 0, 1, 2, 3) and the minima ones are zero at *θ* = 0° + *n* × 90° (*n* = 0, 1, 2, 3), due to the factor $sin^{2}\left(2\theta \right)$. The Raman intensities have four maxima which locate at *θ* = 0° + *n* × 90° (*n* = 0, 1, 2, 3) for the E_g_ mode (Figure 7c) due to the factor $cos^{2}\left(2\theta \right)$.

**Figure 8:**
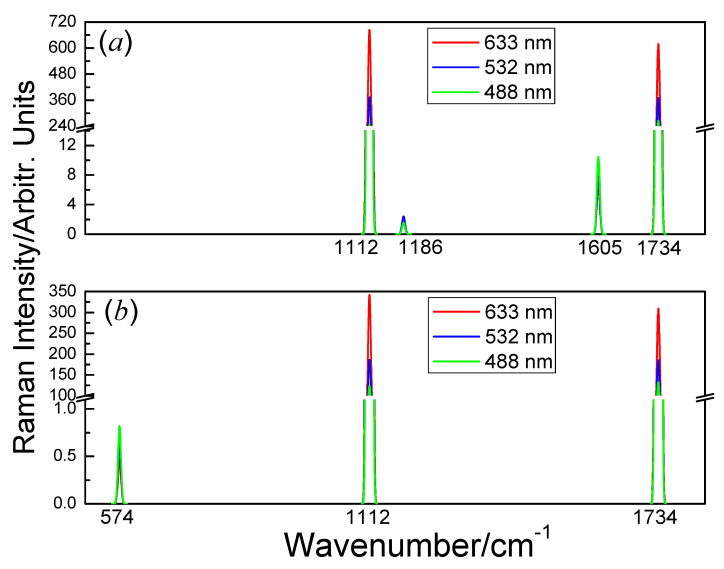
Raman spectra of the octagon-structure carbon monolayer for three laser lines: 488 nm (green), 532 nm (blue) and 633 nm (red) with a Gaussian broadening width of 3 cm^−1^. (**a**) The incident light is from *z*-axis. (**b**) The incident light is from *x*-axis.

By integrating the Raman intensity of all polarization directions, we obtained the monolayer’s nonpolarized Raman intensity of the monolayer when the incident light wave vector is the along *z* and *x* direction (Figure 8). The factor $\omega_{s}^{4}$ in Equation (2) is omitted in Figure 8 for clarity.

When the incident light is along the *z*-axis, the monolayer has four distinct peaks (Figure 8a) at 1112 cm^−1^ (B_1g_), 1186 cm^−1^ (B_2g_), 1605 cm^−1^ (B_2g_) and 1734 cm^−1^ (A_1g_). When the incident light is along the *x*-axis, the monolayer has three peaks (Figure 8b) at 574 cm^−1^ (E_g_), 1112 cm^−1^ (B_1g_) and 1734 cm^−1^ (A_1g_). Whatever the direction of the incident light, the Raman intensity of 1112 cm^−1^ (B_1g_) and 1734 cm^−1^ (A_1g_) are always the strongest and the second strongest.

We calculated the energy of the octagon 2D carbon and compare it with the other allotrope: graphene, graphite and diamond (Table 1). It is found that the octagon carbon monolayer’s energy is greater than graphene and diamond. It means graphene, graphite and diamond are more stable than the octagon 2D carbon.

## 4. Discussion and Conclusions

The Raman shifts for graphene and graphite are 1350, 1600 and 2700 cm^−1^ [53,54]; the Raman shifts for diamond are 1332, 2015 and 2176 cm^−1^ [55]. The strong and distinct 1112 cm^−1^ (B_1g_) and 1734 cm^−1^ (A_1g_) Raman shifts of octogen 2D carbon are unique. They can help materials scientists to distinguish it from graphene, graphite and diamond.

There are five Raman active modes: 574 cm^−1^ (E_g_), 1112 cm^−1^ (B_1g_), 1186 cm^−1^ (B_2g_), 1605 cm^−1^ (B_2g_) and 1734 cm^−1^ (A_1g_). When the incident light wave vector is perpendicular to the plane of the carbon monolayer, B_1g_ and B_2g_ modes can show polarization-angle-dependent Raman intensity. This is different from some other isotropic 2D materials (e.g., *β*-Arsenic monolayer [47], Zr_2_C monolayer and Zr_2_N monolayer [49]). There all are four maxima and minima for all the laser lines when the polarization direction of the scattered light is parallel or perpendicular to that of the incident light. The incident light wave vector is parallel to the plane of the carbon monolayer. For the A_1g_ and B_1g_ modes, Raman intensities have two (four) minima and two (four) maxima when the polarization direction of scattered light is parallel (perpendicular) to that of the incident light. The intensity is zero for the B_2g_ mode. For the E_g_ mode, there always are four minima and four maxima when the polarization direction of the scattered light is parallel or perpendicular to that of the incident light.

The Dirac cone may result in many excellent physical properties, such as very high carrier mobility and an anomalous quantum Hall effect. Due to the two-phonon double resonance Raman process between Dirac points, there will be some double resonant bands whose Raman shifts are approximately twice the Γ point phonon frequencies [54,56].

In conclusion, we predicted a new phase of 2D carbon: 2D octagon-structure carbon. It is found to be semimetal with two Dirac points. We investigated its vibrational properties and polarization resolved Raman spectra excited by three commonly used laser lines 488nm, 532nm and 633nm. The Raman spectra has four and three distinct peaks when the incident light wave vector is perpendicular and parallel to the carbon monolayer, respectively. We expect this octagon-structure carbon can be grown in experiments and would have wide applications.

## Figures and Tables

**Table 1:** Free energy of graphene, graphite, diamond and octagon carbon.

Material	Average Energy/Atom
Graphene	−9.224 eV
Octagon Carbon monolayer	−8.711 eV
Graphite	−9.306 eV
Diamond	−9.099 eV
Octagon Carbon Bulk	−8.782 eV
